# Divergent Cellular and Humoral Immunity to BK Polyomavirus in Healthy Adults and Renal Transplant Recipients Based on Blood and Urine Samples: Implications for Immune-Guided Monitoring

**DOI:** 10.3390/biomedicines14081820

**Published:** 2026-08-13

**Authors:** Deema Ibrahim Fallatah, Steve Christmas

**Affiliations:** 1 College of Applied Medical Science, Prince Sattam bin Abdulaziz University, Al-Kharj 11942, Saudi Arabia; 2 Institute of Infection & Global Health, University of Liverpool, Ronald Ross Building, 8 West Derby Street, Liverpool L69 7BE, UK

**Keywords:** BK polyomavirus (BKPyV), T-cell Immunity, renal transplantation, humoral immune response, viral reactivation

## Abstract

**Background/Objectives**: BK polyomavirus (BKPyV) establishes lifelong, asymptomatic persistence but frequently reactivates in renal transplant recipients, contributing to significant post-transplant morbidity. Although humoral responses are routinely monitored, the role of cellular immunity in viral control remains incompletely characterized. This study aimed to characterize BKPyV-specific T-cell responses and IgG-binding levels in healthy adults and renal transplant recipients and to explore their association with active BKPyV viruria. **Methods**: BKPyV DNA was screened and quantified in urine samples from eighty-two healthy adults and thirty-three renal transplant recipients, who were categorized into active, no, historical, and pre-transplant infection groups. BKPyV-specific cellular immunity was assessed in peripheral blood mononuclear cells using IFN-γ ELISPOT and T-cell proliferation assays, and IgG binding levels were measured in plasma using semi-quantitative ELISA. **Results**: Healthy BKPyV-positive individuals had significantly higher IFN-γ-secreting T-cell frequencies than BKPyV-negative individuals, with no significant difference in IgG levels. Transplant recipients with detectable viruria had markedly diminished IFN-γ responses compared with all other groups, whereas IgG levels remained comparable except in the pre-transplant group (n = 2). A strong negative correlation was observed between IgG levels and urine viral load in actively infected patients. Proliferation assays showed no detectable antigen-specific proliferation despite measurable cytokine secretion. **Conclusions**: Cellular immunity, rather than total binding IgG levels, may be more closely associated with BKPyV activity, particularly in transplant recipients. Given the exploratory cross-sectional design, prospective validation is needed. These findings support integrating cell-mediated immune assays into BKPyV monitoring to improve post-transplant risk assessment.

## 1. Introduction

BK polyomavirus (BKPyV) is a ubiquitous human polyomavirus that remains latent in the urinary tract of most adults but can reactivate and cause clinically significant disease in immunocompromised hosts, particularly kidney transplant recipients. Reactivation may lead to BK-associated nephropathy and graft dysfunction, making BKPyV an important cause of post-transplant morbidity [1].

Controlling BKPyV relies on coordinated cellular and humoral immune responses. Virus-specific T cells, especially IFN-γ-producing CD4+ and CD8+ cells, play a key role by directly killing infected cells and releasing antiviral cytokines [2]. CD4+ T-helper cells are vital for maintaining CD8+ cytotoxic responses and supporting B-cell maturation, while CD8+ T cells directly lyse BKPyV-infected renal epithelial cells [3]. In transplant patients, immunosuppressive drugs mainly weaken these cellular responses, creating conditions favourable for viral reactivation. Measuring these responses through methods like ELISPOT or functional assays can identify patients at risk of ongoing viremia and help optimize immunosuppression [2,3]. Humoral immunity also offers protection; recent studies show that genotype-specific neutralizing antibody levels are linked to a lower risk of post-transplant BK DNAemia. However, total binding IgG levels measured by standard ELISA may not accurately reflect neutralizing ability or clinical prognosis, as binding antibodies don’t necessarily block viral entry [4,5]. This difference is crucial since passively transferred neutralizing antibodies are being investigated as potential treatments [6,7].

Clinical management currently relies mainly on early virologic monitoring and reduction of immunosuppression when BK DNAemia is detected; however, this approach risks precipitating rejection, and there is growing interest in integrating immune monitoring to guide individualized interventions. Recent work has explored passive immunotherapy (IVIG), adoptive transfer of BKPyV-specific T cells, and the potential use of neutralizing monoclonal antibodies as adjuncts to antiviral strategy, highlighting the translational momentum in the field [6,7].

Despite these advances, gaps remain in understanding how the cellular and humoral arms interact in both healthy carriers and immunosuppressed patients, and how different assay readouts (IFN-γ secretion, proliferation, and neutralization) relate to viral control. Addressing these gaps is critical to developing immune-guided management strategies that balance antiviral control with graft preservation [8,9].

Accordingly, this study compares BKPyV-specific IFN-γ T-cell responses and binding IgG levels between healthy immunocompetent adults and renal transplant recipients across clinical categories of infection status, aiming to clarify how cellular and humoral markers align with detectable viral replication in these populations.

## 2. Materials and Methods

### 2.1. Ethical Approval

Ethical approval was obtained from the University of Liverpool Interventional Ethics Committee. All aspects of the study were conducted in accordance with the Declaration of Helsinki (1975, revised 2000) and relevant institutional guidelines. Written informed consent was obtained from all healthy volunteers and renal transplant recipients before participation. All participants were informed about the purpose of the study, sample collection procedures, potential risks, and their right to withdraw at any time without consequence.

### 2.2. Study Participants

A total of 82 healthy participants were recruited through advertising at the Department of Infection and Global Health. Urine samples were collected from all participants for BKPyV DNA screening. Blood samples (for plasma and PBMC isolation) were collected from all 82 healthy participants for ELISPOT and ELISA analyses, including the 5 participants whose urine samples tested positive for BKPyV DNA and all 77 PCR-negative participants. For the proliferation assay, which was performed as a pilot experiment, blood was collected from a subset of 4 participants (2 BKPyV+ and 2 BKPyV−). For renal transplant patients, participants were recruited from the Liverpool University Hospital. Both urine samples (for BKPyV DNA detection and quantification) and blood samples (for immunological assays) were collected from these participants and stored at −20 °C until analysis. BKPyV DNA was detected exclusively in urine for all participants; plasma viral load was not measured.

### 2.3. Detection and Quantification of BKPyV DNA

BKPyV DNA was detected and quantified exclusively in urine samples for all participants. To extract viral DNA, urine samples (200 µL) were centrifuged at 14,000× *g* for 10 min to obtain cell pellets, which were resuspended in phosphate-buffered saline. Viral DNA was then extracted using the QIAamp viral DNA Mini Kit (Qiagen, Hilden, Germany) according to the manufacturer’s protocol, with a final elution volume of 60 µL. For quantification, a standard curve was prepared using ten-fold serial dilutions of a plasmid containing the BKPyV VP1 gene (range: 10^1^ to 10^6^ copies/µL). qPCR was performed in a final volume of 20 µL containing 10 µL of QuantiTect SYBR Green PCR Master Mix (Qiagen, Hilden, Germany), 0.5 µM each of forward primer (5′-AGTCTTTAGGGTCTTCTACCTTT-3′) and reverse primer (5′-GGTGCCAACCTATGGAACAG-3′), 5 µL of extracted DNA, and nuclease-free water. Thermal cycling conditions were as follows: initial denaturation at 95 °C for 15 min, followed by 40 cycles of 95 °C for 15 s, 58 °C for 30 s, and 72 °C for 30 s. All reactions were performed in duplicate. Viral load in urine samples was calculated by interpolating the cycle threshold (Ct) values against the standard curve, and results were expressed as copies/mL of urine.

### 2.4. BKPyV IgG ELISA

BKPyV-specific IgG levels were measured using a semi-quantitative commercial ELISA kit (Creative Diagnostics, Shirley, NY, USA, DEIASL161; pre-coated with BKPyV VP1 virus-like particles), performed according to the manufacturer’s instructions. All samples were tested in duplicate, and the mean OD values were used for analysis. The kit included positive and negative controls, which served as the calibration standards. The cutoff for seropositivity was calculated as the mean OD of the negative controls plus 2 SD, as per the manufacturer’s protocol. According to the manufacturer, the kit has an intra-assay CV < 10% and inter-assay CV < 15%. A 100 µL volume of diluted plasma sample (1:100) was added to each well, except for those reserved for positive and negative controls, which were added afterward for validation. The plates were then incubated for one hour at room temperature. After incubation, the plates were washed three times with wash buffer; 100 µL of the enzyme-linked secondary antibody (anti-human IgG conjugated to horseradish peroxidase (HRP); Sigma-Aldrich, St. Louis, MO, USA) was then added to each well, and the plates were incubated for 1 h at room temperature before being washed three times with wash buffer. The substrate solution (100 µL) was then added to each well and the plates were incubated for 10–15 min in the dark. The reaction was stopped by adding stop solution to each well, and the optical density was measured at 450 nm using an ELISA plate reader. BKPyV IgG levels are expressed as optical density (OD) values, with results compared against positive and negative controls included on each plate.

### 2.5. Gamma Interferon Enzyme-Linked Immunospot Assay

Peripheral blood mononuclear cells (PBMCs) were isolated from whole blood by density gradient centrifugation using Lymphoprep (Axis-Shield, Oslo, Norway). ELISPOT assays were performed using PVDF-bottom 96-well plates (Merck Millipore, Burlington, MA, USA) pre-coated overnight at 4 °C with an anti-human IFN-γ capture antibody (Mabtech, Nacka Strand, Sweden). Plates were blocked with RPMI-1640 medium containing 10% FBS for 2 h at 37 °C. PBMCs were added at a density of 2.5 × 10^5^ cells per well in duplicate and stimulated with BKPyV overlapping peptide pools (JPT Peptide Technologies, Berlin, Germany) spanning the entire BKPyV proteome (VP1, VP2, VP3, large T-antigen, and small T-antigen), at a final concentration of 1 µg/mL per peptide. Cells were incubated for 18–24 h at 37 °C in 5% CO_2_. Positive controls consisted of cells stimulated with phytohaemagglutinin (PHA; 5 µg/mL; Sigma-Aldrich, St. Louis, MO, USA) and negative controls consisted of unstimulated cells (RPMI-1640 medium alone). After incubation, plates were washed six times with PBS and incubated with biotinylated anti-IFN-γ detection antibody (Mabtech, Nacka Strand, Sweden) for 2 h at room temperature, followed by streptavidin-alkaline phosphatase conjugate (Mabtech, Nacka Strand, Sweden) for 1 h. Spots were developed using BCIP/NBT substrate (Mabtech, Nacka Strand, Sweden) and the reaction was stopped by washing with tap water. Plates were air-dried, and spots were counted using an automated ELISPOT reader (AID Autoimmun Diagnostika, Straßberg, Germany) with AID ELISPOT software version 7.0. Results are expressed as spot-forming units (SFU) per 2.5 × 10^5^ PBMCs. The mean number of spots in unstimulated wells (background) was subtracted from the mean number of spots in stimulated wells for each sample. A positive response was defined as ≥10 SFU/2.5 × 10^5^ PBMCs after background subtraction and had to be at least twice the mean background spot count.

### 2.6. T-Cell Proliferation Assay

Peripheral blood mononuclear cells were isolated and labelled with 5 µM carboxyfluorescein succinimidyl ester (CFSE; Thermo Fisher Scientific, Waltham, MA, USA) in PBS for 8 min at 37 °C, followed by quenching with ice-cold RPMI-1640 medium containing 10% fetal bovine serum (FBS). After labelling, CFSE-stained PBMCs (2 × 10^5^ cells/well) were cultured in 96-well round-bottom plates in RPMI-1640 supplemented with 10% FBS, 100 U/mL penicillin, and 100 µg/mL streptomycin. Cells were stimulated with BKPyV overlapping peptide pools (1 µg/mL; JPT Peptide Technologies, Berlin, Germany) for 5–7 days at 37 °C in 5% CO_2_. Negative controls comprised unstimulated cells, while positive controls were stimulated with anti-CD3/CD28 beads (Dynabeads; Thermo Fisher Scientific, Waltham, MA, USA) at a 1:1 bead-to-cell ratio. Following incubation, cells were harvested, washed with FACS buffer (PBS containing 1% FBS and 0.1% sodium azide), and stained with the following surface antibodies: anti-CD3-APC (clone OKT3, BioLegend, San Diego, CA, USA) and anti-CD4-PE (clone RPA-T4, BioLegend, San Diego, CA, USA). Cells were incubated with antibodies for 30 min at 4 °C in the dark, washed twice, and resuspended in FACS buffer. Data acquisition was performed on a BD FACSCanto II flow cytometer (BD Biosciences, Franklin Lakes, NJ, USA), and a minimum of 20,000 events in the lymphocyte gate were collected per sample. Flow cytometry data were analyzed using FlowJo software (BD Biosciences, Franklin Lakes, NJ, USA). T-cell proliferation was assessed by CFSE dilution within gated CD3+ CD4+ T-cell populations. This pilot experiment was performed on a subset of 4 participants (2 BKPyV+ and 2 BKPyV− healthy participants). Due to the pilot nature of this experiment, proliferation was assessed qualitatively by CFSE dilution rather than by formal proliferation index calculation. Viability data and detailed gating strategies were not systematically recorded. CD8+ T-cell proliferation was not evaluated in this pilot experiment.

### 2.7. Statistical Analysis

Tables and charts were used to display the results. Quantitative variables are reported as mean ± standard deviation (SD) or median with ranges, depending on the nature of the data. For comparisons between two groups, the non-parametric Mann–Whitney U test was used, which does not assume normality and is appropriate for small group sizes. For comparisons involving more than two groups, ANOVA was employed. Statistical significance was defined as *p* < 0.05. Exact *p*-values are reported where applicable. When applicable, correlation analysis was conducted using Spearman’s rank correlation. Individual data points are presented in the relevant figures.

## 3. Results

### BKPyV Screening

**Figure 1:**
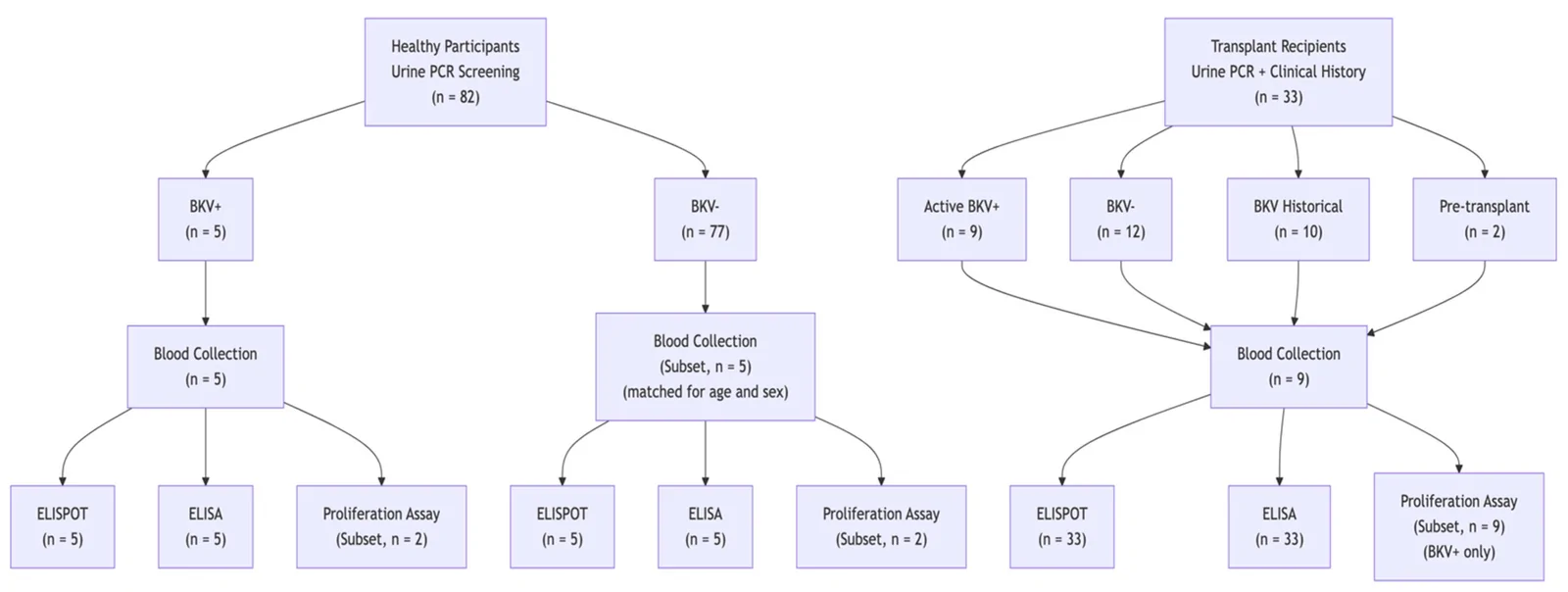
Participant flow diagram showing the number of participants included in each assay. All analyses used the participant as the biological unit.

Healthy participants were screened for BKPyV using urine qPCR. Participants included both males and females, aged 18 to 74. Of these participants, five had detectable BKPyV DNA in urine (defined as >500 copies/mL, i.e., BKPyV viruria), while the remainder had undetectable BKPyV DNA in urine (<500 copies/mL). For healthy participants, classification was based solely on the presence or absence of detectable viruria at the study visit. Among those positive for BKPyV, four of the five were males and had no prior pathological conditions related to BKPyV or renal infections (Table 1). However, one of the male participants positive for BKPyV was on monthly anti-TNF therapy for an arthritic condition. For the post-renal transplant participants, BKPyV infection status was determined by urine qPCR and clinical history. Classification was based exclusively on urine PCR; plasma viral load was not measured. Participants were categorised into four groups: detectable BKPyV viruria (BKPyV+; *n* = 9), defined as detectable BKPyV DNA in urine at >500 copies/mL (viruria); BKPyV-negative (BKPyV−; *n* = 12), defined as undetectable BKPyV DNA in urine (<500 copies/mL) with no prior history and no documented history of BKPyV infection in their medical records; historical BKPyV infection (BKPyV historical; n = 10), defined as documented past infection with subsequent viral clearance (undetectable in urine at sampling); and pre-transplant infection (Pre-tx; *n* = 2), defined as documented infection before transplantation. Blood and urine samples were collected on the same day for each participant, ensuring that virological and immune testing were performed on samples obtained at the same visit. Some pathological conditions reported by the transplant recipients include polycystic kidney, obstructive uropathy, and glomerulonephritis. One transplant recipient presented for re-transplantation (Table 2) (Figure 1).

**Figure 2:**
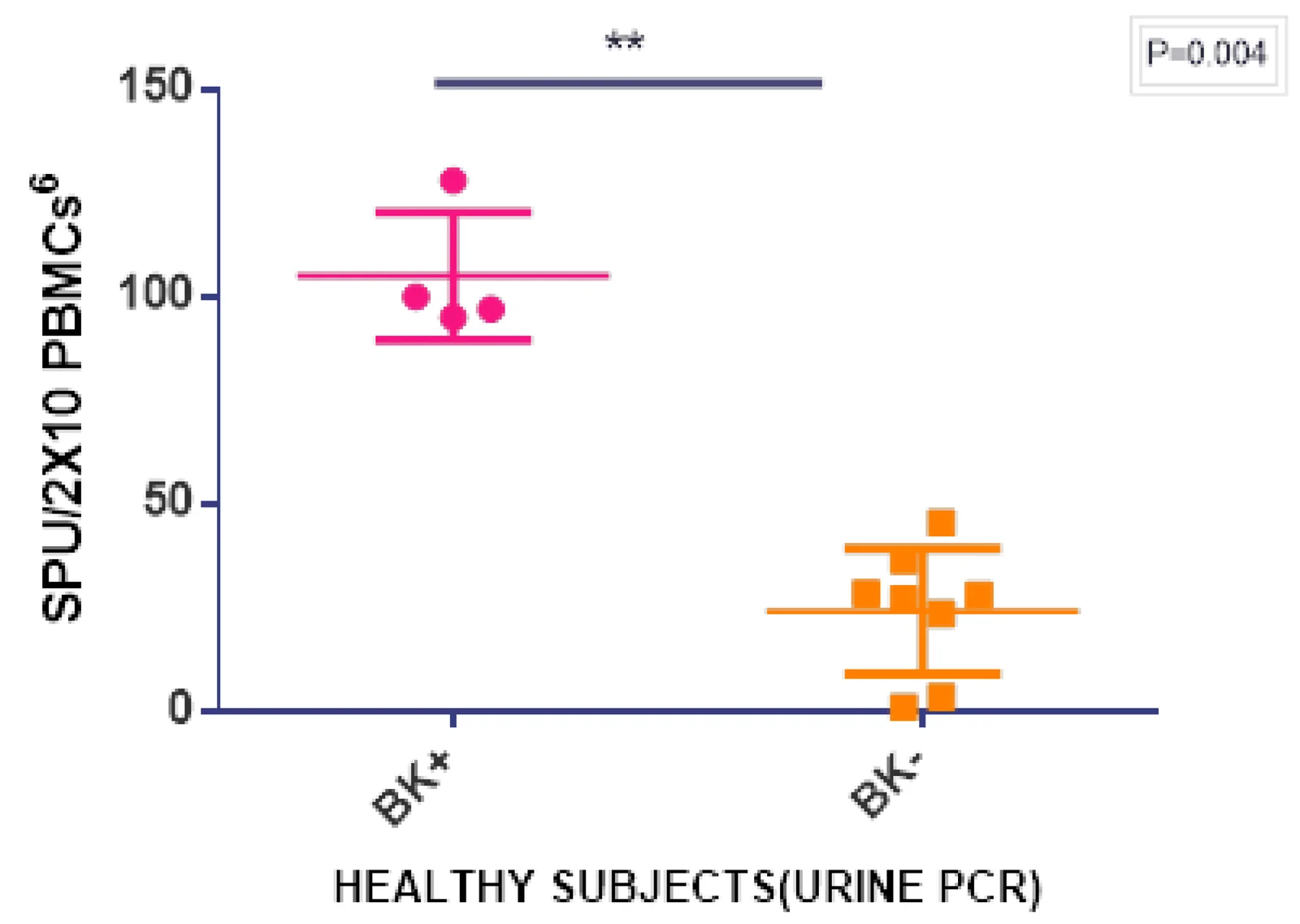
BKV-specific cellular immune response in healthy volunteers. Frequency of BKV-specific IFN-γ secreting cells in healthy volunteers with (BKV+, n = 5) or without (BKV−, n = 77) active BKV infection, as measured by ELISPOT assay following stimulation with BKV overlapping peptide pools. Bars represent the mean ± standard error of the mean (SEM). Statistical significance was determined by the Mann-Whitney U test (*p* = 0.036).

**Figure 3:**
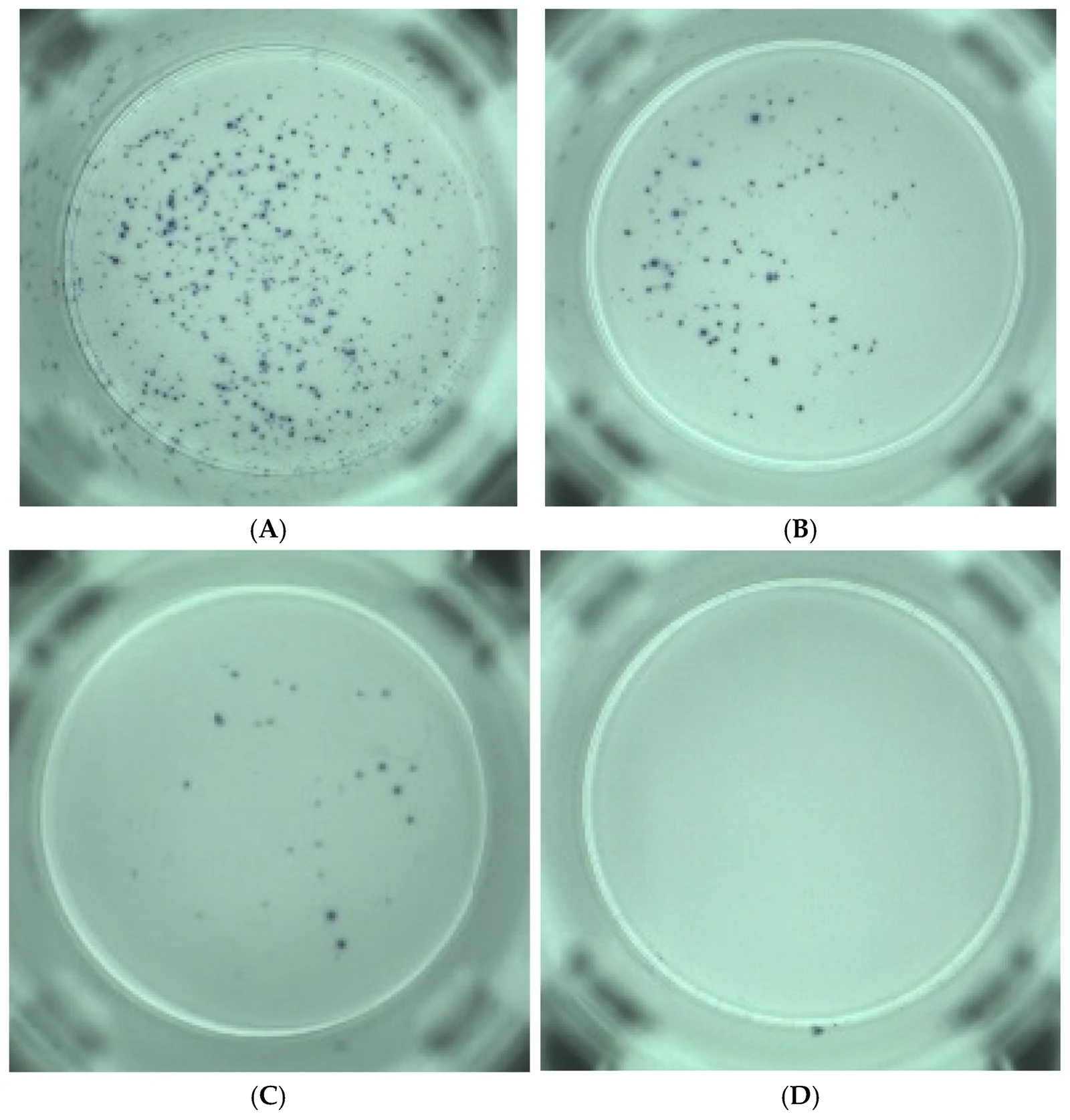
Representative ELISPOT wells from healthy participants. Visual examples of ELISPOT wells demonstrating IFN-γ spots, each representing a reactive T-cell. (**A**) A BKV-positive subject showing a strong response. (**B**) Positive control (anti-CD3 stimulation) showing robust T-cell activation. (**C**) A BKV-negative subject showing no specific response. (**D**) Background (unstimulated) control well.

A detailed analysis of the adaptive immune response to BKPyV was conducted in healthy immunocompetent individuals. To evaluate the cell-mediated immune response, peripheral blood mononuclear cells (PBMCs) were isolated from the whole blood of BKPyV-positive (BKPyV+) and BKPyV-negative (BKPyV−) participants. These cells were subsequently stimulated ex vivo with overlapping peptide pools spanning the major BKPyV proteins (VP1, VP2, VP3, large T-antigen, and small T-antigen). The frequency of BKPyV-specific T-cells was quantified using an Enzyme-Linked Immunospot (ELISPOT) assay, which detects the secretion of interferon-gamma (IFN-γ), a key effector cytokine of antiviral cellular immunity. This analysis revealed a statistically significant difference in cellular responses between the two groups (*p* = 0.036, Mann–Whitney U test; Figure 2). However, we caution that the BKPyV+ group consisted of only 5 participants compared to 77 in the BKPyV− group, and this finding should be interpreted as exploratory and requires validation in larger, more balanced cohorts. The qualitative appearance of these responses is demonstrated by representative ELISPOT wells, which show distinct spots corresponding to individual reactive T-cells in a BKPyV-positive subject, in contrast to the clear wells of a BKPyV-negative subject and background controls (Figure 3).

**Figure 4:**
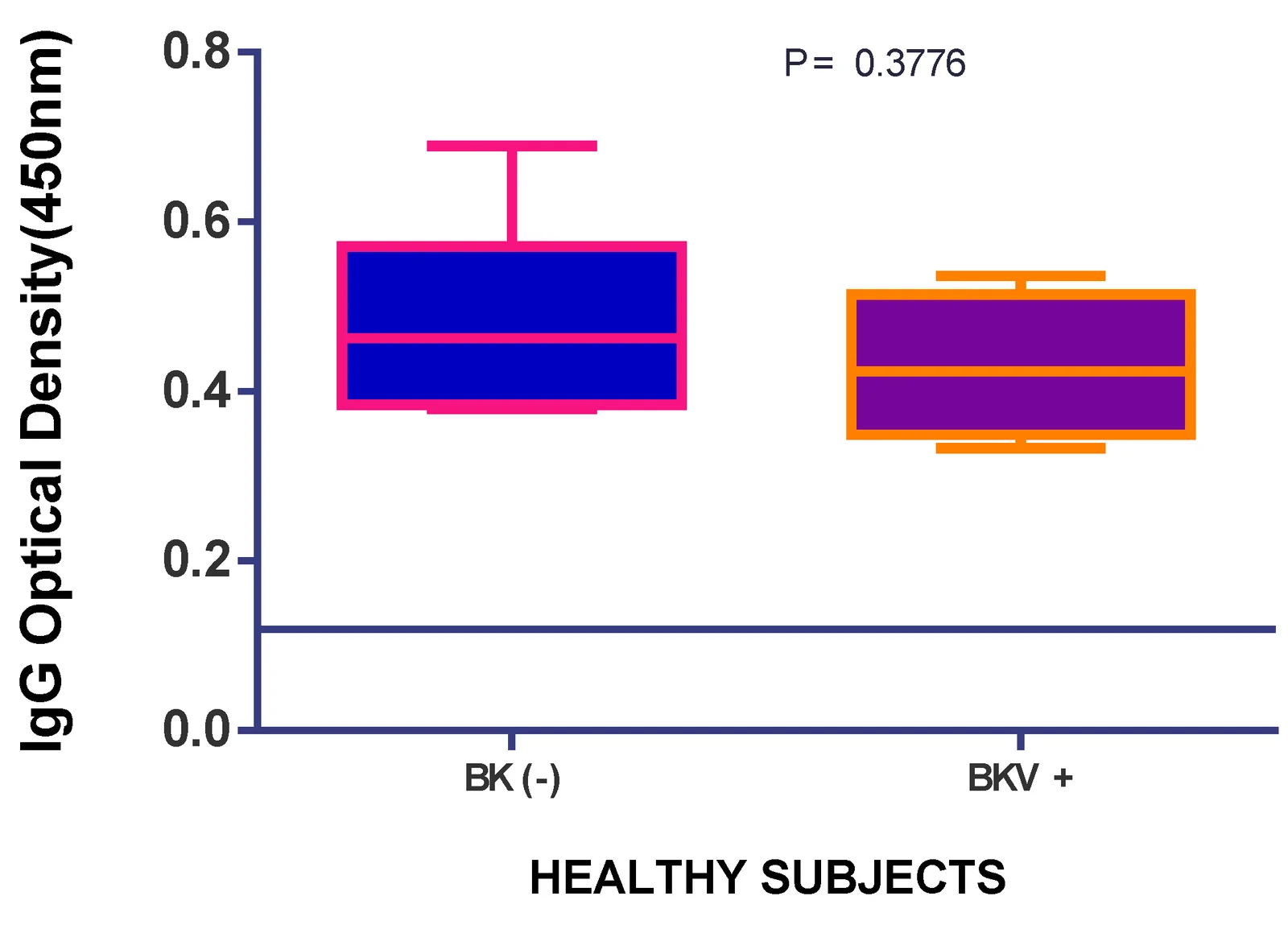
BKV-specific humoral immune response in healthy volunteers. BKV-specific IgG antibody levels in plasma samples from healthy BKV+ (n = 5) and BKV− (n = 77) participants, measured by ELISA. Antibody levels are expressed as optical density (OD) at 450 nm. The box and whiskers plot shows the 10th to 90th percentiles. The difference between groups was not statistically significant (*p* = 0.37).

**Figure 5:**
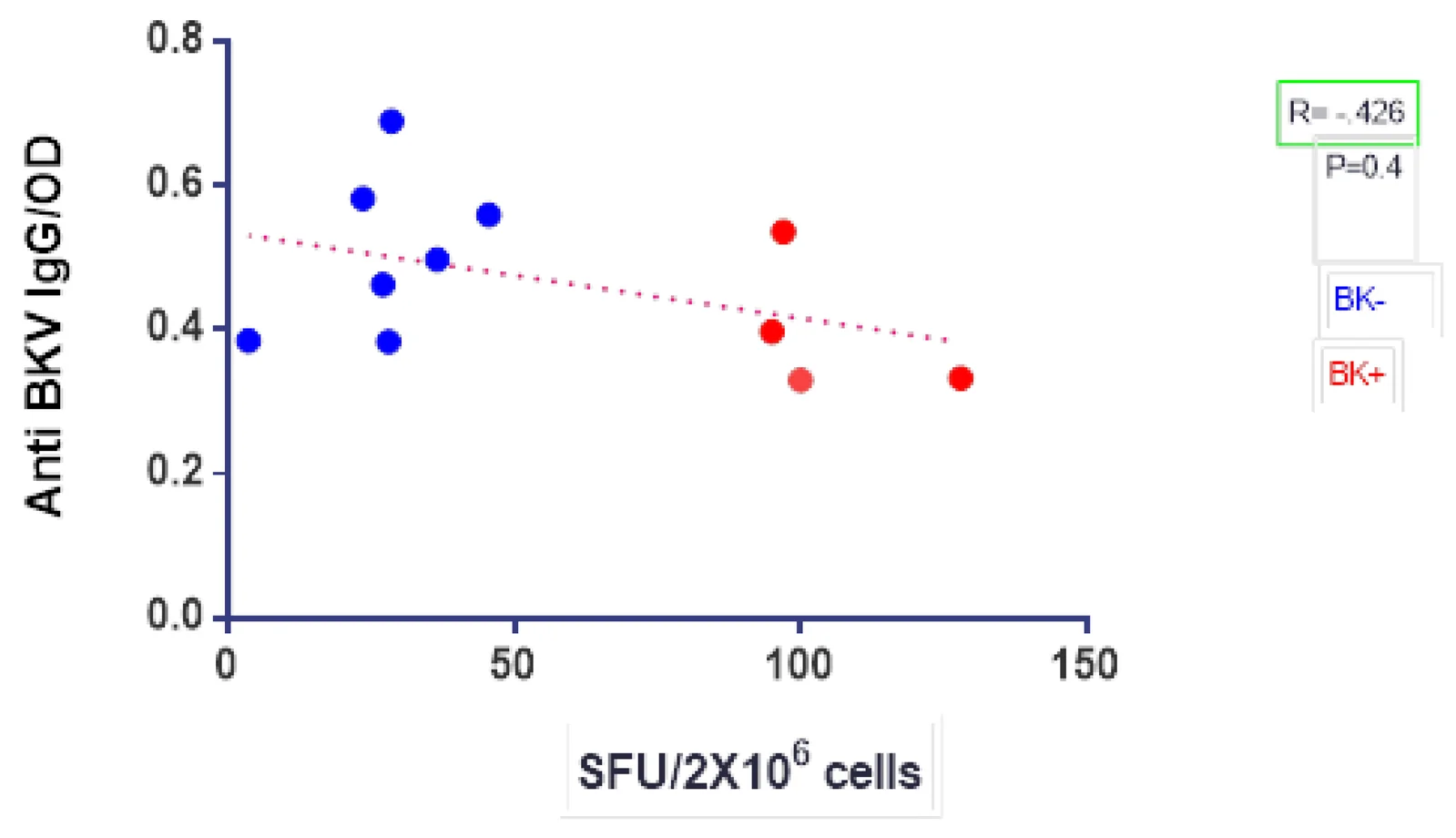
Correlation between cellular and humoral immune responses to BKV in healthy participants. Scatter plot showing the relationship between the number of BKV-specific IFN-γ secreting cells (SFU/2.5 × 10^5^ PBMCs) and BKV-specific IgG levels (OD 450 nm) for each healthy participant. A slight, non-significant negative correlation was observed, as calculated by Spearman’s correlation.

In parallel, BKPyV-specific binding IgG levels were measured in plasma using a semi-quantitative ELISA. Seropositivity was determined based on optical density (OD) values relative to a calculated cut-off. Contrary to the cellular response trend, the level of BKPyV-specific IgG was slightly lower in the BKPyV+ group compared to the BKPyV- group. However, this difference in antibody levels did not reach statistical significance (*p* = 0.37; Figure 4), indicating that in this cohort of healthy individuals, active viral shedding was not associated with a heightened antibody titer. Given the divergent trends between cellular responses and binding IgG levels, we investigated the potential relationship between these two components of the adaptive immune response. A correlation analysis was performed, plotting the number of IFN-γ-secreting cells against the corresponding BKPyV-specific IgG OD value for each participant. This analysis revealed a slight, but not statistically significant, negative correlation (Figure 5). This trend suggests that in healthy carriers, there may be an inverse relationship between the magnitude of the T-cell response and the circulating antibody level. However, this requires validation in a larger cohort.

**Figure 6:**
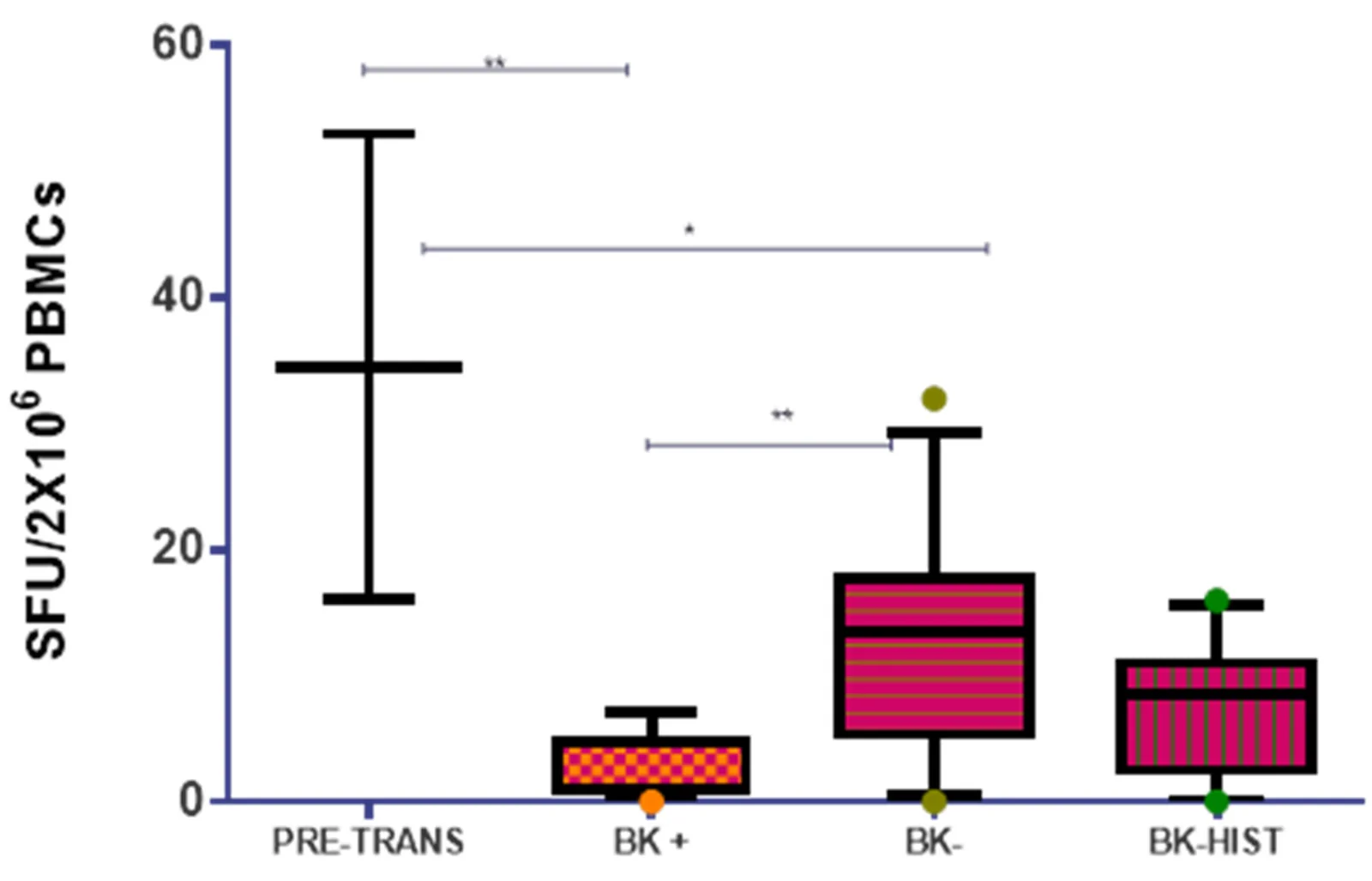
BKV BKV-specific cellular immune response in renal transplant patient groups. IFN-γ ELISPOT responses across the four groups of renal transplant recipients: patients with active BKV infection (BKV+, n = 9), no history of infection (BKV−, n = 12), past resolved infection (BKV Historical, n = 10), and pre-transplant patients with prior infection (Pre-tx, n = 2). Data is presented as spot-forming units (SFU) per 2.5 × 10^5^ PBMCs. For comparisons between groups, ANOVA was used as a global test with post-hoc comparisons where appropriate. The BKPyV+ group showed significantly lower responses compared to all other groups (*p* < 0.05 for each comparison).

The immune response to BKPyV was next characterized in a cohort of renal transplant recipients, a population at high risk for viral reactivation and disease. Patients were stratified into four distinct clinical categories based on their BKPyV infection history and current status: active infection (BKPyV+), no history of infection (BKPyV−), past resolved infection (BKPyV historical), and pre-transplant patients with a prior infection. Analysis of the cellular immune response using an ELISPOT assay revealed striking differences between these groups. The patient group with detectable BKPyV viruria exhibited the lowest frequency of BKPyV-specific IFN-γ secreting cells (Figure 6). This blunted cellular response stands in stark contrast to the robust response observed in the BKPyV group, which demonstrated the highest IFN-γ production. The BKPyV historical and pre-transplant groups showed intermediate yet significantly higher cellular responses than the actively infected group. This pattern indicates that detectable BKPyV viruria in the context of transplantation and immunosuppression is associated with a deficient virus-specific T-cell response.

**Figure 7:**
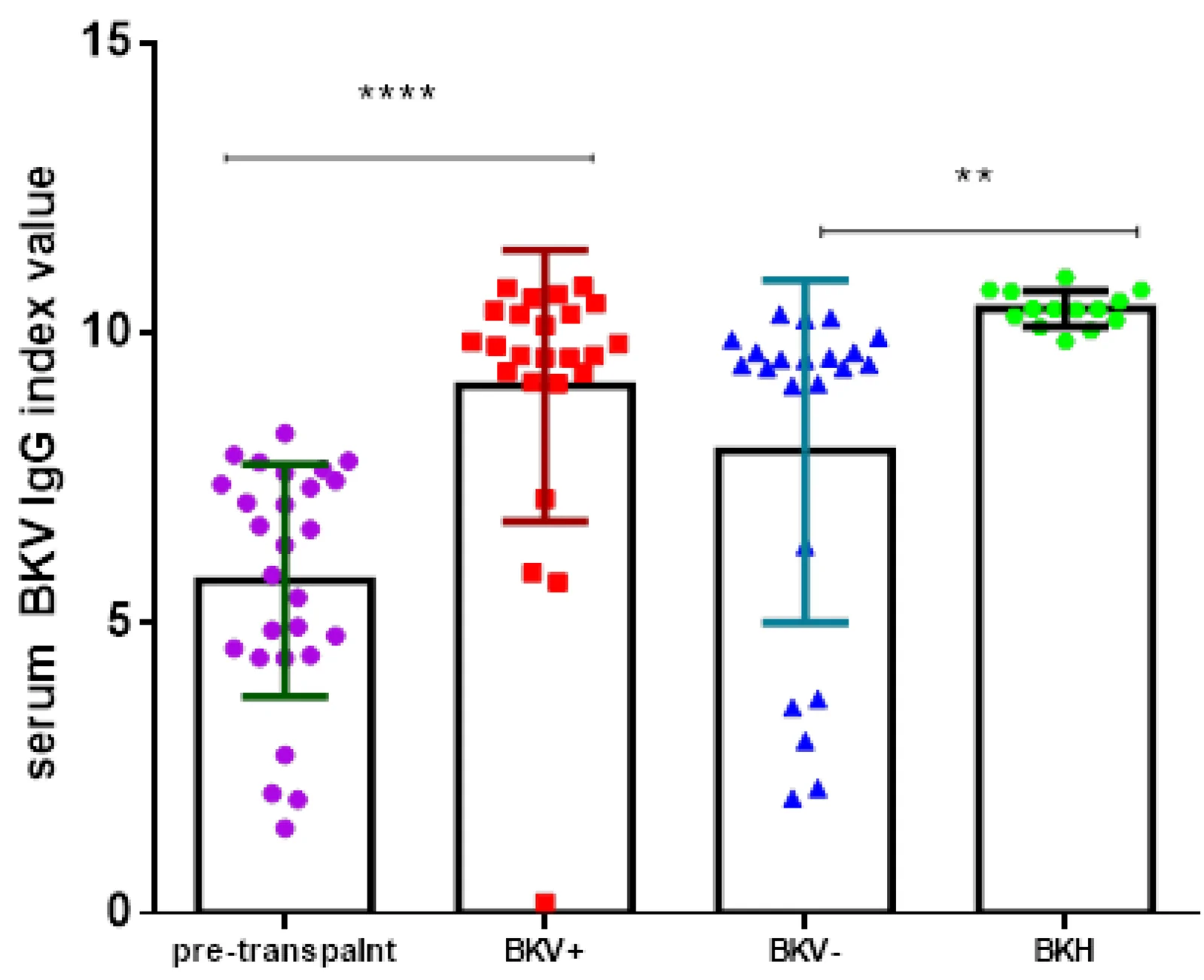
BKV-specific humoral immune response across renal transplant patient groups. BKV-specific humoral immune response across renal transplant patient groups. BKV-specific IgG levels measured by ELISA across the four renal transplant recipient groups. Antibody levels are expressed as OD 450 nm. Each point represents an individual participant (biological unit); technical replicates were averaged per participant. The pre-transplant group (Pre-tx; n = 2) is shown for descriptive purposes only; statistical comparisons involving this group were not performed due to small sample size. Comparisons among the BKV+, BKV−, and BKV Historical groups showed no significant differences.

**Figure 8:**
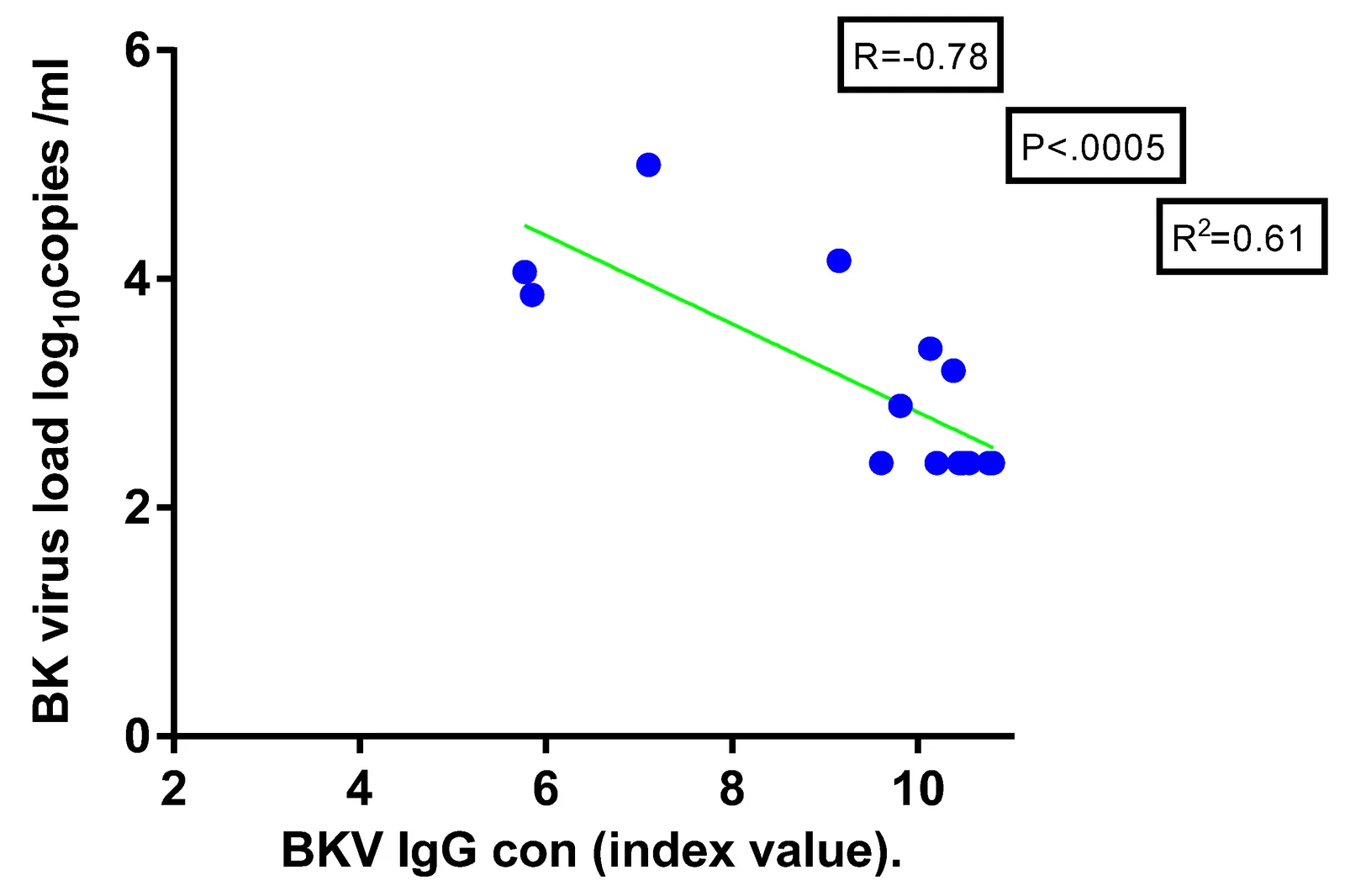
Correlation between BKV-specific IgG and viral load in patients with detectable BKPyV viruria. Scatter plot illustrating the relationship between BKV-specific IgG levels (OD 450 nm) and BKV urine viral load (copies/mL) in renal transplant recipients with detectable BKPyV viruria (n = 9). A negative correlation was observed (Spearman’s r = −0.7, *p* = 0.03).

The BKPyV-specific binding IgG profile across the transplant groups presented a different pattern. Measurement of BKPyV-specific IgG revealed that the pre-transplant group (*n* = 2) appeared to have lower average antibody levels compared to the other three groups; however, no statistical comparisons were performed involving this group due to the small sample size (Figure 7). The BKPyV+, BKPyV−, and BKPyV historical groups all maintained comparably high levels of BKPyV-specific IgG, with no significant differences among them. This suggests that while immunosuppression may not uniformly suppress antibody levels, the pre-transplant state or the timing of infection may influence humoral immunity differently. A critical analysis was performed to understand the interplay between binding IgG levels and viral activity in patients with active infection. We correlated the BKPyV-specific IgG levels with the corresponding viral load. This analysis yielded a negative correlation (*r* = −0.7, *p* < 0.05, Figure 8). This finding indicates that within the actively infected transplant group, higher viral loads are associated with lower BKPyV-specific antibody levels, potentially reflecting an inability of the humoral immune system to control viral replication or antibody consumption due to high antigenic load.

**Figure 9:**
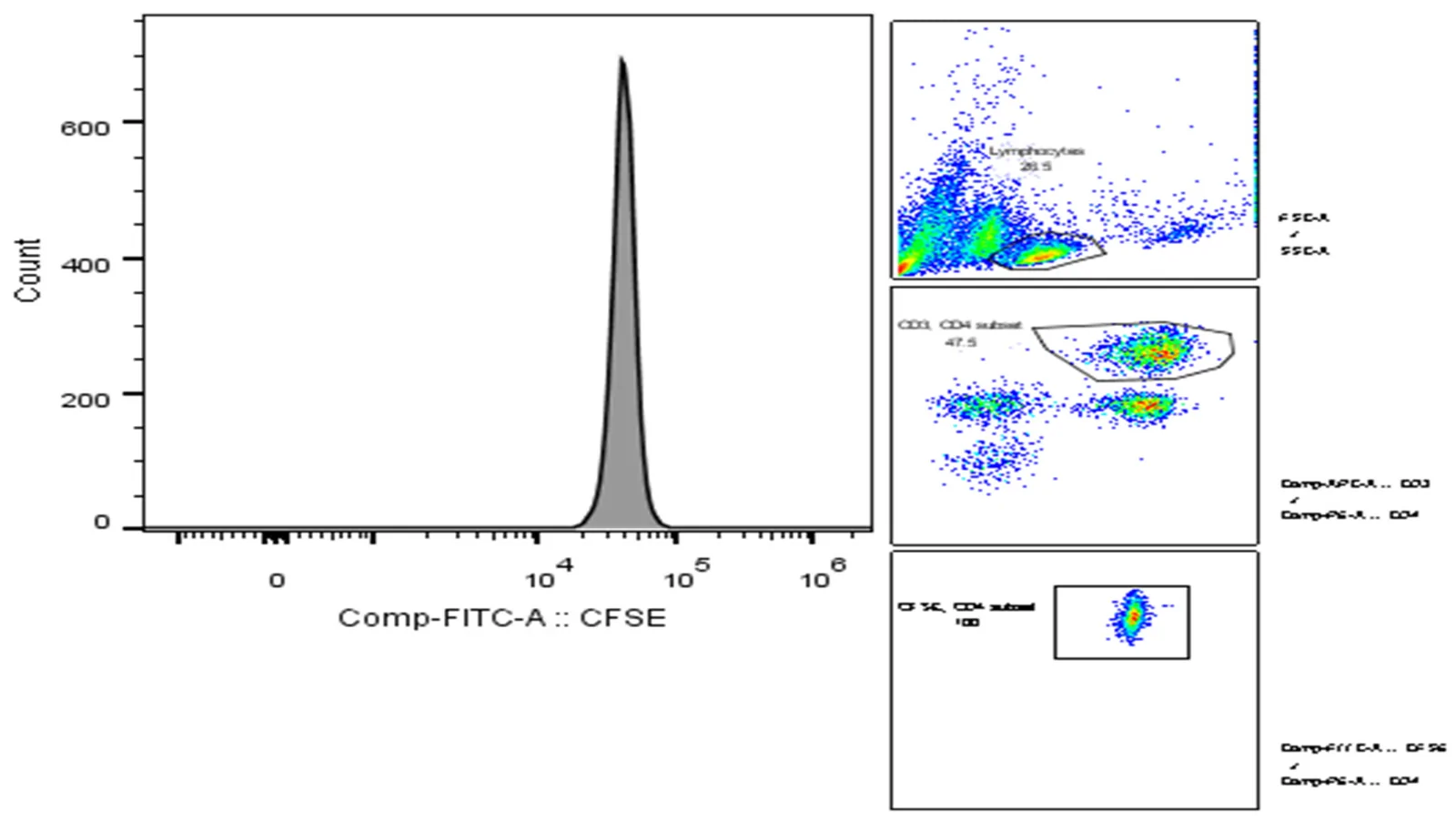

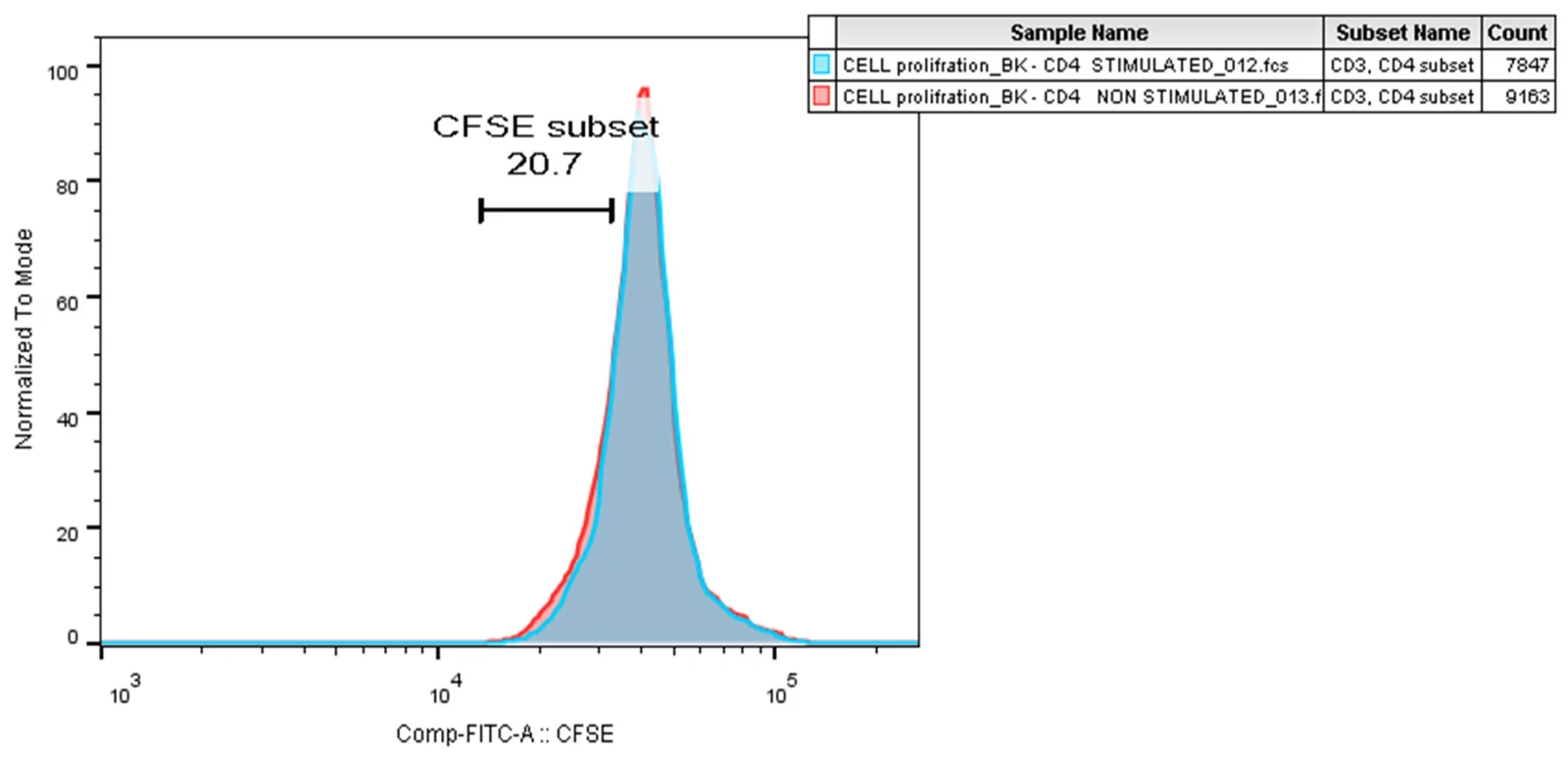
Assessment of antigen-specific T-cell proliferation in response to BKV peptides (representative experiment from 4 participants). Lymphocyte proliferation assay measured by CFSE dilution via flow cytometry. Histograms show CFSE intensity in gated CD3+CD4+ T cells. The blue peaks represent unstimulated cells, and the red peaks represent cells stimulated with BKV peptides for 5–7 days. The overlapping peaks indicate no observable antigen-specific T-cell proliferation in response to BKV peptide stimulation in this pilot experiment.

To further investigate the nature of the T-cell response, a complementary functional assay was employed. A lymphocyte proliferation assay was designed to directly measure the capacity of BKPyV-specific T-cells to expand upon encountering viral antigens. This pilot experiment was performed on a subset of 4 participants (2 BKPyV+ and 2 BKPyV− healthy participants). PBMCs were labelled with carboxyfluorescein succinimidyl ester (CFSE) and cultured for five to seven days in the presence or absence of BKPyV peptide pools. Anti-CD3/CD28-stimulated cells served as positive controls and demonstrated robust proliferation in all experiments, confirming that the assay conditions were functional. Following the culture period, proliferation was assessed by flow cytometry within gated CD3+CD4+ T cells. The resulting histograms showed complete overlap between the peaks of unstimulated (blue) and BKPyV peptide-stimulated (red) CD3+CD4+ T cells (Figure 9). The absence of a distinct peak of CFSE-low cells in the stimulated samples suggests that no measurable antigen-specific proliferation occurred under these experimental conditions. This observation was made in a limited number of samples as a pilot experiment; therefore, no formal proliferation indices or viability data were calculated. The lack of proliferation in CD4+ T cells, despite the detection of IFN-γ secretion in the ELISPOT assay, may indicate that the responding T cells in these participants are terminally differentiated effector cells with limited proliferative capacity, or that the in vitro culture conditions were suboptimal for inducing proliferation. However, given the pilot nature of this experiment and the fact that exhaustion or differentiation phenotypes were not directly measured, we interpret this finding with caution. CD8+ T-cell proliferation was not evaluated.

## 4. Discussion

This study characterizes cellular and binding IgG responses to BKPyV in healthy individuals and renal transplant recipients. In healthy participants, detectable BKPyV infection was associated with strong IFN-γ-secreting T-cell responses by ELISPOT, consistent with reports that BKPyV-specific T cells can be generated and expanded in vitro for adoptive therapy in immunocompromised patients [10]. This reinforces the idea that even in immunocompetent carriers, cell-mediated immunity remains a key mechanism for controlling BKPyV replication. We acknowledge, however, that the healthy BKPyV+ group consisted of only 5 participants compared to 77 BKPyV− participants, representing a major limitation of this comparison. While the observed difference was statistically significant, the small and unbalanced sample sizes limit the generalizability of this finding. Accordingly, we interpret these results as exploratory and hypothesis-generating, and emphasize that validation in larger, more balanced cohorts is essential. We note that the transplant recipient cohort in this study partially overlaps with our previous publication on anti-BKPyV IgG [11]. However, the present study includes novel analyses not previously reported, including ELISPOT assays measuring BKPyV-specific IFN-γ-secreting T-cell responses, T-cell proliferation assays, correlation analyses between cellular and humoral responses, and correlation between IgG levels and urine viral load.

Contrasting with cellular immunity, BKPyV-specific IgG levels in healthy BKPyV-positive individuals were slightly but non-significantly lower than in BKPyV-negative ones, and a non-significant negative correlation existed between IFN-γ responses and IgG OD. For healthy participants, BKPyV-negative status was based on a single negative urine qPCR at the time of screening, without prior medical history. Given that BKPyV seroprevalence in healthy adults exceeds 80%, some individuals classified as BKPyV-negative by urine PCR may nevertheless have had previous exposure and latent infection [1]. This limitation may have contributed to the lack of significant differences in IgG levels between the groups. This suggests that total binding IgG levels (as measured by ELISA) may not reliably reflect recent viral activity or immune control. Indeed, recent literature has emphasized that measuring neutralizing antibodies—rather than total IgG—is more informative for assessing protective humoral immunity [11,12]. In sum, our observations argue against overinterpreting modest ELISA IgG differences as evidence of effective or ineffective viral control.

In the transplant cohort, renal transplant recipients with detectable BKPyV viruria (detectable BKPyV DNA in urine) exhibited the lowest BKPyV-specific T-cell responses. In contrast, those without detectable viruria or with resolved infection showed higher responses. This aligns with recent data indicating the key role of functional T-cell immunity in preventing or controlling BKPyV reactivation after transplantation. For instance, a 2024 study demonstrated that kidney transplant recipients with BKPyV-associated nephropathy had significantly impaired BKPyV-specific T-cell proliferation, cytokine secretion, and cytotoxic functions, often with an exhausted T-cell phenotype [13]. Our findings support this association: a deficient cellular response is associated with active viral replication among immunosuppressed recipients. While post-transplant BKPyV monitoring remains the standard of care, our findings raise the question of whether pre-transplant assessment of BKPyV-specific cellular and humoral immunity could help stratify patients at risk for post-transplant reactivation. However, we acknowledge that this remains a debatable topic as the evidence base is currently insufficient to support routine clinical implementation. Prospective studies are needed to determine whether pre-transplant immune profiling can guide individualized immunosuppression strategies that balance antiviral control with graft preservation.

Regarding binding IgG responses, we observed that the two pre-transplant patients appeared to have lower IgG levels than post-transplant patients. However, no statistical inference can be drawn from this small group. In patients with active infection, IgG levels inversely correlated with urine viral load. This supports recent studies showing that pre-transplant binding antibody levels are poor predictors of post-transplant BKPyV reactivation [12]. Furthermore, some studies have shown that even in the presence of antibodies, a lack of effective cellular immunity may permit viral replication and nephropathy [11,14]. These parallels suggest that binding IgG level alone is insufficient to guarantee viral control in the transplant context. We emphasize that observations involving the pre-transplant group are exploratory and hypothesis-generating and require validation in larger cohorts.

Finally, our observation that antigen-specific CD4+ T cells did not proliferate in vitro despite IFN-γ secretion should be interpreted with caution as this was a pilot experiment performed on a limited number of samples (4 participants) without formal proliferation index calculation. The anti-CD3/CD28 positive control confirmed assay functionality, but CD8+ T-cell proliferation was not evaluated, and the lack of proliferation data in this subset is a limitation. The finding mirrors patterns reported in post-transplant BKPyV infection cohorts, in which cytokine-producing T cells often exhibit limited proliferation or cytotoxic capacity [8,13]. One speculative possibility is that this reflects a predominance of differentiated or exhausted effector T cells; however, we emphasize that exhaustion or differentiation phenotypes were not directly measured in this study, and alternative explanations such as suboptimal culture conditions or immunosuppressive effects cannot be excluded. We acknowledge that additional optimization and validation of the proliferation assay would be required before definitive conclusions can be drawn. The study reveals differences between functional readouts (cytokine secretion vs. proliferation) and highlights the need for assays that combine multiple functional measures. We acknowledge that detailed data on specific immunosuppressive regimens were not collected; however, all transplant recipients were on standard triple therapy (calcineurin inhibitor, mycophenolate, and corticosteroids) as per institutional protocol. Future studies incorporating detailed drug type, dosage, and trough levels would help delineate drug-specific effects on anti-BKPyV immunity. Furthermore, systematic renal biopsy data to confirm BKPyV-associated nephropathy were not available for all actively infected patients as biopsies are only performed when clinically indicated. Future studies should include histopathological correlation with BKPyV nephropathy to better link immune responses with clinical outcomes.

## 5. Conclusions

The study supports the finding that, in healthy individuals, cellular immune responses to BKPyV are detectable and robust, whereas binding IgG level alone poorly reflects active infection. In the transplant setting, impaired BKPyV-specific T-cell function, rather than antibody levels, appears more tightly associated with BKPyV viruria. These findings suggest that cellular immune monitoring may have value in assessing BKPyV infection and caution against overreliance on binding-IgG measurements alone for clinical decisions. However, given the exploratory and cross-sectional nature of this study, these conclusions require validation in larger, prospective cohorts before any clinical implementation can be recommended. Future studies should incorporate neutralizing antibody assays and longitudinal sampling to clarify causal relationships between immune responses, viral load, and clinical outcome.

## Figures and Tables

**Table 1:** Baseline characteristics of healthy participants.

Variable	BKPyV+	BKPyV−
N	5	77
Mean Age (years)	48	34
Gender—male, female	4, 1	40, 37
Pathological conditions	1	0

**Table 2:** Baseline characteristics and pathological conditions of renal transplant patients.

Variable	Total	BKPyV+	BKPyV−	BKPyV Historical	Pre-Transplant	*p* Value
N	33	9	12	10	2	
Mean Age (years)	53.7	55.6	60	58.3	41	NS
Gender—male, female	26, 7	9, 0	7, 5	8, 2	2	NS
Serum creatinine (mean) (µmol/L)	219	197	136	158	385	NS
Re-transplantation, n (%)	1	1 (11)	-	-	-	
Polycystic kidney, n (%)	7	3	3	1	-	NS
Obstructive uropathy, n (%)	2	-	2	-	-	NS
Glomerulonephritis, n (%)	5	4	-	1	-	NS
Other, n (%)	17	2	7	8	-	NS
Rejection, n (%)	1	1				

Detectable BKPyV viruria was defined as urine viral load >500 copies/mL; BKPyV− was defined as undetectable viral load (<500 copies/mL) with no prior history; historical defined as documented past infection with subsequent clearance (undetectable at sampling); pre-transplant was defined as documented infection before transplantation. NS, not significant.

## Data Availability

The original contributions presented in this study are included in the article. Further inquiries can be directed to the corresponding author.
